# The casual effect of lifestyle factors on outcomes of assisted reproductive techniques: a protocol study on Iranian infertile couples

**DOI:** 10.1186/s12978-018-0655-8

**Published:** 2018-12-17

**Authors:** Mahdi Sepidarkish, Reza Omani-Samani, Mohammad Ali Mansournia, Mir Saeed Yekaninejad, Azar Mardi-Mamaghani, Samira Vesali, Roya Hosseini, Saharnaz Nedjat

**Affiliations:** 1 Department of Epidemiology and Biostatistics, School of Public Health, Tehran University of Medical Sciences, P.O. Box: 16635148, Tehran, Iran; 2 Department of Epidemiology and Reproductive Health, Reproductive Epidemiology Research Center, Royan Institute for Reproductive Biomedicine, ACECR, Tehran, Iran; 3 Department of Andrology, Reproductive Biomedicine Research Center, Royan Institute for Reproductive Biomedicine, ACECR, Tehran, Iran

**Keywords:** Reproductive techniques, Life style, Cohort studies, Causality, Iran

## Abstract

**Background:**

Attention to additional factors affecting the success of assisted reproduction techniques (ARTs) is very important and with appropriate interventions in some of these factors, success rate can be as high as improve a lot. There is a lot of evidence that lifestyle factors can influence on ARTs outcomes. Current knowledge of the effect of combined effect of several lifestyle factors on the outcomes of ARTs is low and contradictory. The importance of the causality of this phenomenon is felt by the pandemic of inappropriate lifestyle as well as the dramatic increase in infertility in the world. The aim of this cohort study is to scrutinize the casual effect of a specific range of contemporary lifestyle factors on ARTs outcomes.

**Methods:**

A prospective cohort study will be conducted in Royan institute, Tehran, Iran. Each infertile couple will be monitored from the first visit to the end of treatment cycle. The data will be collected electronically and include the following: detailed interview about lifestyle and socioeconomic status, past medical history, general physical examination, assessment of menstrual and ovulatory status, assessment of tuboperitoneal, assessment of uterine, assessment of cervix, urogenital examination, semen analysis, anti-sperm antibodies, biochemical analysis, sperm-cervical mucus contact test, in vitro cervical mucus penetration test and sperm functional assays. To estimate the casual effect of lifestyle variables on clinical pregnancy and live birth, the obtained propensity score (PS) from generalized boosted models (GBM) will be matched between couple with and without live birth.

**Discussion:**

This is the first study to prospectively obtain detailed information on causes of ARTs success. Determining the casual effect of lifestyle variables on ARTs success will be important to inform strategies most likely to increase the success rates in ARTs.

## Plain English summary

Based on 17 annual reports of the European in vitro fertilization (IVF) Monitoring Consortium (EIM) between 1997 and 2013, the clinical pregnancy rate following ARTs was relatively stable. Even in infertile couples who have known reasons for their infertility and receive treatment according to their problem, success rates, as measured by the live birth, is noticeably low. However, evidence has shown that even in couples whose infertility is unexplained, by choosing the appropriate treatment approach, the chance of success is up to 70%. The first basic step in treatment of infertile couples is counseling them to modify their inappropriate lifestyle. To date, few studies have examined the casual effect of lifestyle factors on success rate among infertile couples. In this prospective cohort study the causative effect of lifestyle factors on clinical pregnancy and live birth will be examined by matching the obtained PS from GBM between couple with and without live birth. The data will be collected electronically and include the following: detailed interview about lifestyle and socioeconomic status, past medical history, general physical examination, assessment of menstrual and ovulatory status, assessment of tuboperitoneal, assessment of uterine, assessment of cervix, urogenital examination, semen analysis, anti-sperm antibodies, biochemical analysis, sperm-cervical mucus contact test, in vitro cervical mucus penetration test and sperm functional assays.

## Background

According to the World Health Organization (WHO) definition, infertility is a disability in pregnancy after one year of normal sexual intercourse and without prevention [1]. It is estimated that about 50 to 80 million people around the world experience infertility during their reproductive life [2]. Based on a systematic analysis of 277 demographic and reproductive health surveys, in 2010, among women 20 to 44 years old who were exposed to the risk of pregnancy, 1.9% (95% CI: 1.7 to 2.2%) were unable to achieve a live birth. Also among women who had a living child and were exposed to the risk of pregnancy, 10.5% (95% CI: 9.5 to 11.7%) were unable to have another child. In parallel with the population growth, the absolute number of infertile couples has also increased. The number of infertile couples increased from 42.0 million (95% CI: 39.6 million to 44.8 million) in 1990 to 48.5 million (95% CI: 45.0 million to 52.6 million) in 2010. It is expected to keep growing, and estimates have put the total infertile couple at 60 million by mid-2030 [3]. Over the past three decades, many cases of infertility have resulted in pregnancy through the modern fertility treatments. There are several types of ARTs, that are used depend on the cause of infertility in couples [4]. Based on 17 annual reports of the EIM between 1997 and 2013, the clinical pregnancy rates per aspiration and per transfer were relatively stable. For IVF, the mean pregnancy rate per transfer is now 34.5% compared with 27.7% in 1999. For intracytoplasmic sperm injection (ICSI), it is 32.9% compared with 27.9% in 1999 [5, 6]. In spite of some positive changes in the past 20 years, the success rate is still dramatically low. The ultimate goal for treating infertile couple with ARTs is to maximize the number of patients delivering living child. Although these techniques have led to an increase infertility success significantly, attention to additional factors affecting the success of ARTs is also very important and with appropriate interventions in some of these factors, success rate can be as high as improve a lot [7]. Many studies have been done on the factors influencing the outcomes of infertility treatments and the fundamental role of factors such as female age, number of oocytes transferred, quality of sperm, overweight and obesity has been proven [8–11]. Lifestyle factors are the modifiable behaviours and circumstances of life that can greatly contribute to overall health and subfertility. The impact of lifestyle on ARTs outcomes may vary depending on person aetiology and circumstances. There is a lot of evidence that lifestyle factors can influence on ARTs outcomes [12, 13]. For example, studies have demonstrated that female age [14], smoking [15], weight [16], diet [17], exercise [18], psychological stress [19], caffeine consumption [20], alcohol consumption [20] and exposure to environmental pollutants [21] significantly decreases the chance of clinical pregnancy and live birth. Little has been done on the effects of male factors on the outcome of ARTs, and clinical observations in this field are small and contradictory. Several studies have shown that overweight and obesity in men lead to changes in hormone levels, and these changes are shown not only in the levels of testosterone and estrogen, but also on sex-hormone binding [22]. There is strong evidence of the adverse effects of smoking on ARTs outcomes through a range of pathways in both in women and in infertile men. There is a strong association between the number of smoking years during the women’s life time and her risk of not conceiving through IVF [15]. Current knowledge of the effect of combined effect of several lifestyle factors on the outcomes of ART is low and contradictory. The importance of the causality of this phenomenon is felt by the pandemic of inappropriate lifestyle as well as the dramatic increase in infertility in the world. The aim of this cohort study is to scrutinize the impact of a specific range of contemporary lifestyle factors on ARTs outcomes. The cohort study focuses on the non-communicable aetiology for ARTs outcomes associated with potentially interventable lifestyle factors. These factors include tobacco usage, drugs, weight, physical activity, alcohol drinking and caffeine consumption.

### Objectives

1. Determining the causative effect of body mass index on clinical pregnancy and live birth following ARTs.
2. Determining the causative effect of cigarette smoking on clinical pregnancy and live birth following ARTs.
3. Determining the causative effect of water pipe (shisha) smoking on clinical pregnancy and live birth following ARTs.
4. Determining the causative effect of drugs on clinical pregnancy and live birth following ARTs.
5. Determining the causative effect of alcohol drinking on clinical pregnancy and live birth following ARTs.
6. Determining the causative effect of physical activity on clinical pregnancy and live birth following ARTs.
7. Determining the causative effect of caffeine consumption on clinical pregnancy and live birth following ARTs.

## Methods/design

### Study design and setting

This is a prospective cohort study, which conducted in Royan institute, Tehran, Iran. Royan Institute is a public, referral institute which is affiliated to Academic Center for Education, Culture and Research (ACECR). It was established in 1991 as a research institute for Reproductive Biomedicine and infertility treatments. The Institute is the largest infertility treatment center in the country and more than 5000 couples visit the research center annually.

### Ethical considerations

The protocol for recruitment and collection of baseline data and biological sample for the cohort was reviewed and approved by the Royan Institute Research Ethics Committee. All participants will be asked to sign an explicit written informed consent to the use of the data.

### Participants

All couples referring to the institute in 2018 form the study population. Each infertile couple is monitored from the first visit to the end of treatment cycle (infertility treatment outcome). Inclusion criteria are as follows: 1) Age of a woman between 18 and 40 years, 2) Couple’s age between 20 to 55 years. Also, if there is one or more following criteria, the participants will be excluded from the study: 1) The presence of systemic or chronic illness in wife or husband, 2) Use of synthetic drugs in couples, 3) Taking anabolizing drugs in couples, 4) Use of immunosuppressive drugs.

### Recruitment process and data collection

**Fig. 1:**
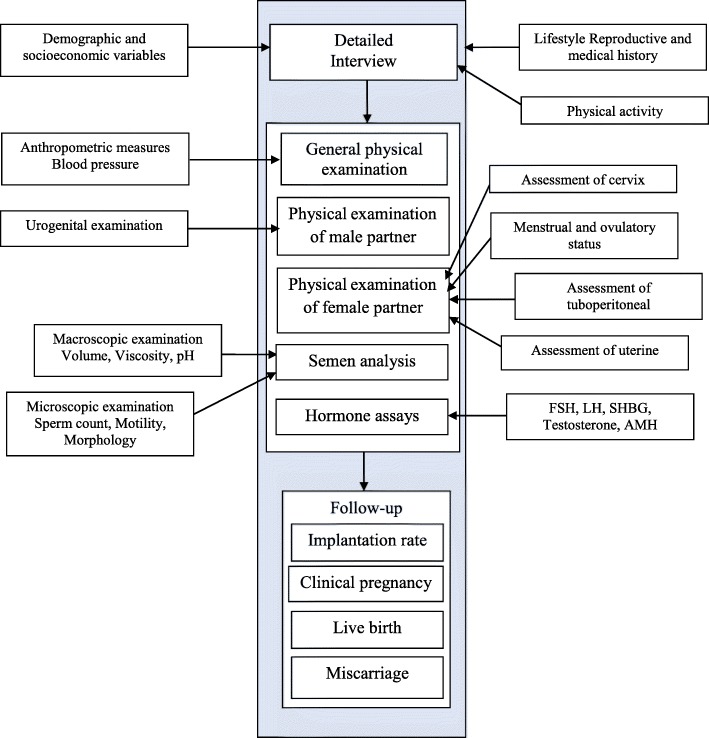
Data gathering process from baseline to the end of follow up

Data collection is done electronically and will be collected at the beginning of the referral of infertile couples. The main framework for data collection is shown in Fig. 1. For infertile women the process will begin with a detailed interview and will continue with general physical examination (including anthropometric measures and blood pressure measurement), assessment of menstrual and ovulatory status, assessment of tuboperitoneal, assessment of uterine and assessment of cervix. For infertile men the process will begin with a detailed interview and will continue with general physical examination (including anthropometric measures and blood pressure measurement), urogenital examination, semen analysis, anti-sperm antibodies, biochemical analysis, sperm-cervical mucus contact test, in vitro cervical mucus penetration test and sperm functional assays. All infertile couple referred to the Royan institute for reproductive biomedicine, after reading and signing informed written consent, will be referred to trained interviewers. The infertile couple will be interviewed by trained interviewers to obtain demographic variables and to complete a 140-item questionnaire regarding lifestyle behavior (smoking habits, alcohol drinking, drug consumption, physical activity), socioeconomic status (occupation, education, income), past medical history, familial history of infertility, reproductive history and assessment of physical activity. Sampling process will continue 12 months (from 1.1.2018 to 1.1.2019). Ten interviewers will collect data electronically in all days of a week (except for holidays). Interviewer bias and quality of data are checked weekly. Independent inspectors regularly look in to 10% of the data each month during the study and data quality.

### Demographic variables of infertile couples

We defined education as follows: the maximum years of education that a person has completed successfully and will be categorized into 3 groups: 1) Illiterate/primary school, 2) Cycle/high school/diploma and 3) Higher than diploma. Economic status of infertile couple will be measured based on ‘asset-based measures’, in which the infertile couple will be asked about ownership of a range of durable assets, housing characteristics and access to basic services [23]. Man occupation will be categorized in two groups as follows: 1) High risk occupation (regarding reproduction and fertility) and 2) Normal occupation. Women occupation will be categorized in two groups as follows: 1) Housewife and 2) Employed.

### Lifestyle variables

We defined the smoking as follows: smoking ≥1 cigarette per day for more than six months continuously. Smoking habits will be categorized in 4 groups: 1) Current smoker, 2) Former smoker, 3) Passive smoker and 4) Never smoked. Also the number of cigarettes smoked a day will be recorded. Water pipe (shisha) smoking will be recorded just like smoking. Drug consumption was defined as repeated use of any psychoactive substance including opioid (opium and morphine derivatives), cannabinoids, cocaine, amphetamines, hallucinogens and multiple drug use for the preceding 6 months. At the first visit, consumption of drug and its frequency was categorized in four categories: 1) None, 2) Monthly (maximum three times per month), 3) Weekly (at least once a week but not every day), and 4) Daily. For alcohol drinking, infertile couple will be asked to explain their drinking status as follows: 1) None, 2) Monthly (maximum three times per month), 3) Weekly (at least once a week but not every day), and 4) Daily. Also type of beverages and average amount will be recorded. The frequency of coffee or tea consumption will be categorized into 4 categories: 1) None, 2) 1–2 cups/day, 3) 3–5 cups/day and 4) More than 5 cups/day.

### Physical activity

The International Physical Activity Questionnaire IPAQ is used to subjectively collects information on the time (number of days and average time per day) spent being physically active across a comprehensive set of domains and measures vigorous physical activities (refer to activities that take hard physical effort and make the person breathe much harder than normal(, moderate physical activity (refer to activities that take moderate physical effort and make you breathe somewhat harder than normal), walking activity (including at work and at home, walking to travel from place to place, and any other walking that you have done solely for recreation, sport, exercise, or leisure), and sitting (including time spent at work, at home, while doing course work and during leisure time) in the last seven consecutive day period [24, 25]. These physical activity information (number of days and average time per day) will be converted to Metabolic Equivalent of Tasks (METs). METs or metabolic equivalent, is a physiological measure expressing the energy cost of physical activities and is defined as the ratio of metabolic rate during a specific physical activity to a reference metabolic rate. One MET reflects the amount of energy consumed while sitting quietly at rest and is equivalent to 3.5 ml/kg/min of VO2 Max [26].

### Past medical and reproductive history

A female past history of reproduction including parity, gravidity, stillbirth, abortion, will be asked in previous pregnancies. Gravidity defined as follows: the number of times that a woman has been pregnant [1]. Parity refers to the previous pregnancies terminating in viable gestational age (20 or more weeks’ gestation) [1]. Stillbirth defined as delivery of an infant with no sign of life at 20 weeks’ gestation or later [1]. Spontaneous abortion or miscarriage refers to spontaneous loss of a fetus prior to 20 weeks [1]. Infertility data including infertility history, infertility duration, cause of infertility, type of infertility, use of fertility treatments, history of donate of gametes or embryos will be collected by interviewers. Cause of infertility in men will be categorized in to seven groups: 1) Immune infertility (antisperm antibodies), 2) Sexually transmitted infections, 3) Genetic, 4) DNA damage, 5) Hypothalamic-pituitary factors, 6) General factors (diabetes mellitus, thyroid disorders), 7) Environmental factors (toxins such as glues, volatile organic solvents or silicones, physical agents, chemical dusts, and pesticides). Cause of infertility in women will be categorized in to seven groups: 1) ovulation problems (e.g. polycystic ovarian syndrome), 2) Tubal blockage, 3) Pelvic inflammatory disease caused by infections like tuberculosis, 4) Age-related factors, 5) Previous tubal ligation, 6) Endometriosis, 7) Advanced maternal age and 8) Immune infertility. Type of infertility will be categorized as: 1) Primary infertility (refers to couples who have not become pregnant after at least 1 year having sex without using birth control methods), and 2) Secondary infertility (refers to couples who have been able to get pregnant at least once, but now are unable). The history of the disease in infertile couple as well as the history of disease in first-degree relatives will also be measured. Desired diseases are as following: diabetes, hypertension, tuberculosis, kidney disease, asthma, heart disease, thyroid disease, pulmonary disease. The infertile woman will be asked about menstrual history including cycle length, duration, and amount of bleeding and premenstrual symptoms. We defined amenorrhoea as follow: the absence of a menstrual period in a woman of reproductive age. It will categorized in two groups: 1) Primary amenorrhoea (meaning a woman never developed menstrual periods), 2) Secondary amenorrhoea (absence of menstrual periods in a woman who was previously menstruating). Dysmenorrhea refers to the occurrence of painful cramps during menstruation. Oligomenorrhea considered as cyclic menstrual bleeding occurring at intervals greater than 35 days, but less than 6 months. Polymenorrhea described as a menstrual cycle that is shorter than 21 days. Menorrhagia defined as “heavy” or “prolonged” menstrual cycle periods (over 80 ml of blood in one cycle). Poly cystic ovary syndrome, will be diagnosed based on two criteria of Rotterdam’s criteria as follows: 1) Oligoovulation and/or anovulation, 2) Excess androgen activity, 3) Polycystic ovaries (by gynecologic ultrasound).

### Physical examination

In the first visit, weight, waist circumference, hip circumference of the couples will be recorded by two nutritionists. Two measurements will be made from each specified part and the average will be recorded. If the second measurement is more or less than a standard deviation from the first measurement, the third measurement will be performed and the median of the measurements will be recorded. The female’s weight with the Scale Seca 813 Digital Scale will be made with the minimum dress. This balance can hold up to 200 kg and a precision of 100 g. Before weighing, the participant is requested to put on his shoes and additional clothes, and do not have anything in his pocket. Height will be measured in the stand position by International Standards for Anthropometric Assessment (ISAK) standard. Height in the stand position without shoes and with a precision of one millimeter in two visits will be measured by the stadiometer company Seca 206. The mean of two measurements will be recorded. If two measurements differ more than 1.5%, the third measurement will be performed and the median measurement will be recorded. Blood pressure is measured using the scorpion pressure gauge by Arkka Switch Germany from the right hand, two times in sitting position and the mean is recorded. The hand is at the same level of the heart and the person has the right support. At least half an hour before blood pressure measurement, the examiner should not have severe activity; heavy food, coffee, alcohol, medicines and stimulants, or smoked. Meanwhile, one should not be fasting for more than 14 h. Five minutes before blood pressure measurement, the examiner should have a full rest. The clothes of an examiner should be sufficiently light. The sleeves of clothing are wide enough. As long as the sleeve is raised and do not push on the arm. The examination room should be quiet and at a suitable temperature. After completion of the general physical examination (anthropometric measures and blood pressure measurement), the gynecologist will examine menstrual and ovulatory status, the tuboperitoneal factor (by hysterosalpingography), uterine (by hysterogram and endovaginal ultrasonography) and cervical factors. The clinical examination of the female reproductive tract will include the following: 1) Uterine fibroids or an ovarian mass, 2) Acute or chronic pelvic inflammatory disease, 3) Ovarian cyst or endometriosis, 4) An abnormal discharge, 5) Bimanual palpation, 6) Uterine fibroids or adenomyosis. Physical examination of the infertile male will be performed by the andrologist. The examination will be focus on penis size, circumcision, the presence of surgical or traumatic scars, the degree of secondary sexual development, induration plaques of Peyronie’s disease, testis size, the presence of a hydrocele and varicocele. Varicocele defined as abnormal ventricular dilatation and torsion of the vein over the testicles. It graded according to the following criteria: Grade III: the distended venous plexus visibly bulges through the scrotal skin; Grade II: there is intrascrotal venous distension which is easily palpable but not visible; and Grade I: distension is only palpable while the patient is performing a Valsalva maneuver.

### Semen analysis (SA)

The semen specimen will be obtained by masturbation into an ID marked, sterile, preweighed, wide-mouth plastic container according to WHO 2010 recommendations (5th edition, WHO, Switzerland) [27]. The infertile couple will be trained to avoid sexual activity 48 to 72 h before the sample is collected. The specimen container will be kept at ambient temperature, between 20 and 37 °C. The sample will be delivered immediately (within 1 h of collection) to the central laboratory on the same floor and incubated in a 378 C incubator. The duration of liquefying will be recorded. The normal reference range of semen parameters was that defined by WHO in 1992. Considering the fact that semen specimen is affected by intra and inter-individual variations, quality evaluation double-checked by two andrologists. If the semen analysis will be abnormal, a repeat analysis will be carried out 2 to 3 months later. Sperm parameters will be assessed include the number of sperm cells, viability, motility, and morphology of the sperm population. The SA will involve two essential parts: 1) Macroscopic examination and 2) Microscopic examination. Macroscopic examination include volume, viscosity and pH. According to WHO standards the average amount of seminal fluid is about 1.5 to 5.5 ml and will be measured by pouring the specimen in to a calibrated cylinder. The viscosity of semen will be evaluated in a cylindrical cylinder. The normal semen will be dropped as a drop in the drip, without any accumulation or adhesion. If viscosity is abnormal, the drop will form a thread more than 2 cm long. The viscosity will be documented in degrees from zero (watery) to 4 (gel). The liquefaction time will be documented in minutes. The average pH of semen should range between 7.2 and 7.8 which is measured by pH meters. Microscopic examination include sperm count, motility and morphology. Sperm count and sperm motility will be assessed by computer-aided sperm analysis (CASA System; Microptic S.L., Barcelona, Spain). The following steps will be performed to evaluate the number of sperm: Initially, the appropriate dilution and proper chambers will be determined by accurate evaluation of the undiluted semen and well-mixed liquefied semen. In the next step, the semen will be mixed and the diluent will be prepared with a fixative. Then haemocytometer chamber will be load and spermatozoa will be replaced in wet chambers. Samples will be evaluated between 10 and 15 min. At least 200 spermatozoa will be counted in each iteration. The concentration will be calculated per ml. Ultimately all spermatozoa per ejaculate will be calculated. Based on WHO recommendation, we will use 100-μm-deep haemocytometer chambers.The dilution and sperm count will be performed twice. The following steps will be performed to evaluate sperm motility: Initially, the semen sample must be mixed well. An aliquot of semen fluid should be removed immediately. A wet preparation with a depth of 20 μm must be prepared for each repetition. The slide must be evaluate with phase-contrast optics at × 200 or × 400 magnification. At least 200 spermatozoa will be counted in each iteration. The motility of each spermatozoon will be categorized in three groups as following: 1) Progressive motility, 2) Non-progressive motility and 3) Immotility. The following steps will be performed to evaluate sperm morphology: Initially, a smear of semen must be prepared on slide. The slide must be dried, fixed and stained. The slide must be evaluated with bright field optics at × 1000 magnification. At least 200 spermatozoa will be assessed in each iteration.

### Hormone assays

Blood samples will be obtained between 07:00 and 09:00 after an overnight fast of at least 12 h between days 3 and 7 of the spontaneous menstrual cycle. All samples will be centrifuged 30 to 45 min after collection and stored at − 70 C until hormone analyses will be performed. (Izotop, Budapest, Hungary, Gamma couter: Dream Gamma- 10, Goyang-si, Gyeonggi-do, South Korea) with the following detection limits: FSH, 0.08 mIU/mL; LH, 0.02 mIU/mL; SHBG and T, 0.04 ng/mL. Intra-assay and interassay CVs are 1.4, 2.8, 2.9 and 2.5%, for FSH, LH, SHBG, and T respectively.

### Primary and secondary outcomes

#### Live birth

Live birth refers to a case where the fetus shows signs of life regardless of the gestational age after leaving the mother’s body. Regardless of whether the cord is cut off or not, or whether it is separated or not. These symptoms can be muscle movement, heart rate, pulse or breathing, even if these symptoms are momentarily and then discontinued [1].

#### Clinical pregnancy

Observing the gestational sac with hearing a fetal heartbeat with ultrasound for at least 5 weeks after embryo transfer [1].

#### Implantation rate

The number of gestational sacs observed at echographic screening at 6 weeks of pregnancy divided by the number of embryos transferred [1].

#### Miscarriage

Natural death of an embryo or fetus before it is viable and especially between the 12th and 20th weeks of gestation [1].

#### Data quality and data management

To ensure the validity and reliability of collected data, a range of strategies will be utilized. This will include validation checks for consistency and completeness of routinely collected data, training of interviewers in administration of the questionnaires, and training of clinical and project staff in measurement of specific indices. All study interviewers who recruit to the study and administer the baseline questionnaire have initial training in how to explain the study objectives, how to take consent and how to administer the questionnaire using a range of strategies, such as formal training sessions, practical demonstrations and role play. A training manual is used to ensure standardization of instructions and consistency of training standards for study interviewers throughout the life of the project. Once they begin recruitment, each study interviewer has a random sample (10%) of interviews observed by an independent observer on a quarterly basis. A set of interviewer notes is also provided to help standardize explanation of specific issues, and to eliminate ambiguity.

#### Sample size calculation

The study required the enrollment of 1417 patients in each group to have at least 80% power to detect a 5% difference in clinical pregnancy rates between obese infertile and normal subjects.

#### Data analysis

First we will compare the baseline clinical and demographic characteristics between couple with and without live birth. We will used Student’s t-test for continuous variables and the chi-square test for categorical variables. To estimate the casual effect of lifestyle variables on primary and secondary outcomes, the obtained PS from GBM will be matched between couple with and without live birth. The variable selection for GBM will be done based on Brookhart et al., recommendation [28]. The variables that are unrelated to the exposure but related to the outcome should always be included in a PS model. These variables included those listed above as baseline characteristics. The PS is the conditional probability of exposure to outcome conditional on a vector of observed covariates [29]. Rosenbaum and Rubin show that adjustment for the scalar PS is sufficient to remove bias due to all observed covariates [30]. Accurate estimation of PS is a challenging process based on the following reasons: 1) Large numbers of covariates, 2) Sparse data, 3) Uncertain association of observed covariates with exposure [31]. PS weights can be estimated through the following methods: logistic regression, classification and regression trees (CART), pruned CART, and the ensemble methods of bagged CART, random forests, and boosted CART [32]. Previous studies showed that GBM appear to be most promising for use in the context of PS analysis. GBM provided substantially better bias reduction and more consistent 95% CI coverage [33]. Data were analyzed using the R statistical software environment version 2.15.1 (R Foundation for Statistical Computing, Vienna, Austria).

## Discussion

This paper has outlined the protocol of a prospective cohort study evaluating the casual effect of modifiable lifestyles on clinical pregnancy and live birth following ARTs. Many studies have investigated the association between the lifestyle and the outcomes of ARTs. To our knowledge there is no cohort study that investigate the casual effect of modifiable lifestyle on clinical pregnancy and live birth following ARTs. We expect that our study provide valuable evidence regarding casual effect of modifiable lifestyles. By improving these lifestyle factors, live birth increases significantly following ARTs. The main strength of the current study is a comprehensive assessment of the factors affecting the outcome of infertility treatment. These factors include semen parameters, infertile couples’ lifestyle, physical activity of couples, past medical and reproductive history, social and economic status. In the present study, the data will be collected electronically and by trained interviewers, which significantly reduces and minimizes the measurement error. Also, due to the high sample size, it is possible to identify risk factors for rare pregnancy outcomes. The main disadvantages of this study, like other cohort studies, are missing during the follow up of the participants. The loss during follow-up would be minimized by cyberspace (creating virtual groups in virtual networks), preparing and distributing educational pamphlets, the ongoing contact of the research team with the participants. The possible selection bias due to recruiting patients from one infertility treatment center will reduce the generalizability of the findings. Considering the importance of the internal validity of the study and the emphasis of the experts on the internal validity than external validity, it was decided to conduct a study at this referral center.

## Conclusion

Infertility is a widespread problem worldwide, which in addition to infertile couples also has an adverse impact on community level. Since most lifestyle factors are modifiable, by knowing the causative effect of these factors on the results of assisted reproductive, advice should be given to couples to help them to make positive changes that may increase their chances of success and delivering a live baby. Also, based on these casual evidence, we can develop a guideline for lifestyle advice and helping clinicians to implement better pre-conception care.

## Abbreviations

ACECR: Academic center for education, culture and research
AMH: Anti-Mullerian hormone
ARTs: Assisted reproduction techniques
CART: Classification and regression trees
CV: Coefficient of variation
EIA: Enzyme-immunometric assay
EIM: European IVF monitoring consortium
FSH: Follicle stimulating hormone
GBM: Generalized boosted models
ICSI: Intracytoplasmic sperm injection
IPAQ: International physical activity questionnaire
IRMA: Immunoradiometric assay
IVF: In vitro fertilization
LH: Luteinizing hormone
METs: Metabolic equivalent of tasks
PRL: Prolactin
PS: Propensity score
SA: Semen analysis
SHBG: Sex hormone-binding globulin
T: Testosterone
WHO: World health organization
